# Optimization of change in epicardial fat thickness for obese patients who lost weight via the bariatric surgery method using central composite and Box-Behnken experimental designs

**DOI:** 10.7717/peerj.11831

**Published:** 2021-08-04

**Authors:** Ferhan Elmalı, Mustafa Agâh Tekindal, Cihan Altın, Can Ateş, Varlık Erol

**Affiliations:** 1 Faculty of Medicine, Department of Biostatistics, İzmir Katip Çelebi University, İzmir, Turkey; 2 Cardiology Department, İzmir Medikal Park, İzmir, Turkey; 3 Faculty of Medicine, Department of Biostatistics and Medical Informatics, Aksaray University, Aksaray, Turkey; 4 Department of General Surgery, Medicana International İzmir, İzmir, Turkey

**Keywords:** Response surface method, Central composite design, Box-Behnken design, Bariatric surgery

## Abstract

**Background:**

The aim of this study was to detect the optimal values for Age, Body Mass Index (BMI) and HOMA-IR of obese patients prior to surgery that results in a maximal decrease of visceral fat mass 6 months after bariatric surgery.

**Method:**

In this study, 3^3^ experimental set-ups were designed. This study was approved by Baskent University Medical and Health Sciences Research Board (Approval number: KA16/281). The study data consisted of 40 obese patients who lost weight through the bariatric surgery between February 2015 and December 2016. The values of BMI, Age and HOMA for the obese patients who lost weight through the bariatric surgery were evaluated in three categories and at three levels; the response variable was determined as the Change in Epicardial Fat Thickness (ΔEFT).

**Results:**

As a result of CCD analysis, the optimum ΔEFT = 2.571 was determined when Age = 30.52, BMI = 45.30, and HOMA = 34.62. As a result of the BBD analysis, the optimum ΔEFT = 3.756 was determined, when Age = 38.36, BMI = 63.18, and HOMA = 14.95. The optimum ΔEFT was modeled with Contour and Response Surface plots.

**Conclusion:**

Based on the two surface response models used in our study, the maximal decrease of visceral fat mass as assessed by measuring echography images of epicardial fat thickness can be obtained by bariatric surgery of persons who are between 31 and 38 year old, have a BMI between 45 and 63 kg/m2 and have a HOMA-IR 34 between 15 and 35. Central Composite Design and a Box-Behnken Design of suitable patient data predicted 35 optimal settings of independent variables for the maximal clinical response of an intervention.

## Introduction

Since biological events are under the influence of multiple factors, it is necessary to evaluate the effects of such factors together in any biological event. For this reason, factorial experiments that can examine different levels of multiple factors in tandem and also address the status of a factor at different levels of other factors widely used in the fields of biology and medicine.

Considering the factors that are thought to have an effect on the response variable in the factorial experiments, it is necessary to create an appropriate experimental design and use appropriate statistical analysis methods in order to minimize the experimental error. None of the combinations tried in the a priori experimental design created may be the best. In other words, the combination that provides the highest efficiency can be found among or outside of those tried. Therefore, a large number of factor combinations are needed in the factorial experiments. However, performing experiments like this is both expensive and time-consuming. In addition, as the number of factors increases, it becomes difficult to find the homogeneous experimental materials required to test all combinations. Therefore, statistical methods that do not require performing experiments involving all combinations have been developed to find the most appropriate combination of factors. One of these methods is the response surface method. In this method the model is created with the help of regression analysis. The extent to which the main effect or interaction effect of a factor has an important effect on the values of the response variable is decided by the regression coefficients. The first step in the response surface methods is to determine the factors that are thought to have an effect on the response variable and the [effect] levels they have. These two criteria usually determine the experimental designs create the regression model (Kul, 2004). The main experimental designs most commonly used, which include three-level factors, are the Central Composit and Box-Behnken experimental designs. In these designs, the point belonging to the factor levels providing the highest efficiency is estimated first. Then, with the way to reach where the actual optimum point is, or by using the coefficients of the quadratic [second-order] response surface function of the first experimental results (the steepest ascent method), the point in question (the combination that provides the highest optimization) is tried to be found by making successive experimentals (Altun, 1998). The optimum point of a quadratic model is obtained with the help of Ridge analysis (Mead & Pike, 1975).

Epicardial fat [adipose] tissue (EFT) is a part of visceral adiposity. It has endocrine and paracrine effects and is associated with the metabolic syndrome (MetS). EFT is located between the myocardium and the visceral pericardium (Rosenthal et al., 2012). There is a local interaction between EFT, the coronary arteries and the myocardium beneath it (Iacobellis et al., 2003). EFT is defined as an endocrine and inflammatory organ due to the proatherogen and proinflammatory cytokines it secretes (Rosenthal et al., 2012; Iacobellis et al., 2003; Trayhurn, 2005). The relationships between EFT and the metabolic syndrome, diabetes mellitus, and coronary artery disease have been shown in many studies (Rosenthal et al., 2012; Iacobellis et al., 2003; Trayhurn, 2005). Various imaging methods are used to measure the amount (volume) of EFT (Rosenthal et al., 2012; Iacobellis et al., 2003; Mazurek et al., 2003; Talman et al., 2014). Transthoracic echocardiography (TTE) is the preferred method for the measuring EFT due to its advantages, such as easy accessibility, low cost, no radiation exposure, and simultaneous acquisition of other cardiac parameters (Rosenthal et al., 2012; Iacobellis et al., 2003; Trayhurn, 2005). The correlation of EFT thickness measured by TTE with the epicardial and abdominal visceral fat volume measured by the magnetic resonance imaging was demonstrated using anthropometric and metabolic parameters (Rosenthal et al., 2012; Iacobellis et al., 2003; Eroglu et al., 2009; Trayhurn, 2005).

The increase in obesity prevalence and the lack of success rates of options other than the surgical treatment have brought this surgical treatment to the fore. Bariatric surgical treatment is very effective in rapid weight loss. There are limited studies in the literature that examine the effect of bariatric surgical treatment on the heart in detail (Alexander, 1997; Özler, 2006).

In this study, small Box-Behnken and Central Composite designs were created to determine the optimum level of the Change in Epicardial Fat Thickness (ΔEFT) of patients who have lost weight through the Bariatric Surgery Method. Both designs were used for the quadratic response surface model. When we determined the group in which we optimized the parameters of Age, Body Mass Index (BMI) and HOMA that were very important in bariatric surgery, we tried to obtain the maximum benefit in the ΔEFT for the patients.

There are studies showing that CCD design is superior to BBD design. However, in a clinical experiment, there is no literature in which CCD design was used in this type of study, which has never been thought of before and which we think is a guide in bariatric surgery. Our main motivation is that the findings obtained as a result of two experiments guide the decision-making mechanism of surgeons performing bariatric surgery. For this reason, CCD design cannot be ignored.

## Method

This study was approved by Baskent University Institutional Review Board and Ethics Committee (Approval number: KA16/281). In this research article prepared for journal, we: obtained the data, information and documents in the framework of academic and ethical rules; provided all the information, documents, evaluations and results in accordance with scientific ethics and moral codes; referred to all of the articles used in this study with appropriate references; have not made any changes to the data used and the results. The information and findings specified in this study are original. We declare above mentioned issues and accept all rights losses that may arise.

The study data consisted of 40 obese patients who lost weight through the bariatric surgery between February 2015 and December 2016. The CCD and BBD experimental designs were determined before the study. Patients were constantly enrolled in the respective designs in the specified experimental set-ups. The values of BMI, Age and HOMA for the obese patients who lost weight through the bariatric surgery were evaluated in 3 categories and at 3 levels; the response variable was determined as the ΔEFT.

A experimental design with a first-order model will have a linear structure. However, in experiments, the presence of curvilinearity may occur as a result of the curvature test. In this case, the quadratic [second-order] response surfaces analysis should be used.

(1) \begin{eqnarray*}y={\beta }_{0}+{\beta }_{1}{x}_{1}+{\beta }_{2}{x}_{2}+{\beta }_{11}{x}_{1}^{2}+{\beta }_{22}{x}_{2}^{2}+{\beta }_{12}{x}_{1}{x}_{2}+\varepsilon \end{eqnarray*}

This model is called the quadratic response surface model and has some features (Kul, 2004; Mead & Pike, 1975; Doğan & Okut, 2003);

(i) Each factor must have at least 3 levels.

(ii) The model must have at least 1 + 2k + k (k-1) 2 different parameters. As a result, the experimental design should contain the data obtained from 1 + 2k + k (k-1) 2 different points.

In these experiments, the point at which the dependent variable takes the maximum or minimum value is called the “stationary point” (Güvenç et al., 2006). This point is located in the center of the system shown as ellipses. In some cases, the stationary point in the center shows neither the maximum nor the minimum value. In this case, the stationary point is called the “saddle point” and the system is called the “saddle system”. One of the most important points in the quadratic response surfaces method is the stationary points. 3-D [three dimensional] graphics (the response surface and contour plots) assist to determine these points.

### Calculation of stationary points

The determination of the components in the quadratic response surface method depends on the size of the coefficients given in the regression equation. The steps to be followed for the calculation of stationary points are as follows.

(i) A quadratic response surface model is estimated with the help of the data obtained from the experiment [experimental].

(ii) Partial derivatives are taken for each of the factors included in a model and equalized to zero.

(2) \begin{eqnarray*} \frac{\partial \hat {Y}}{\partial {\chi }_{1}} = \frac{\partial \hat {Y}}{\partial {\chi }_{2}} =...= \frac{\partial \hat {Y}}{\partial {\chi }_{j}} =0\end{eqnarray*}

(iii) Equation (2) system of equations obtained in step (ii) is solved. A value will be obtained for each factor. These values are put into place in the model and the estimated value of dependent variable for the stationary points is obtained.

It is possible to obtain the stationary points with the help of matrices. If the given model is expressed in matrices;

(3) \begin{eqnarray*}\hat {Y}={b}_{1}+{x}^{{^{\prime}}}b+{x}^{{^{\prime}}}\hat {B}\chi \end{eqnarray*}

In Eq. (3); *b*_1_** is the model constant, *b* is estimate of the linear model coefficients, and $\hat {B}$ is the estimate of the quadratic model coefficients.

In addition;

(4) \begin{eqnarray*}{x}^{{^{\prime}}}= \left[ {x}_{1},\ldots ,{x}_{j} \right] \end{eqnarray*}

$\hat {B}$ is a symmetric matrix with *k x k* dimensions.

(5) \begin{eqnarray*}\hat {B}= \left[ \begin{array}{@{}ccccc@{}} \displaystyle {b}_{11}&\displaystyle \frac{1}{2} {b}_{12}&\displaystyle &\displaystyle &\displaystyle \frac{1}{2} {b}_{1q}\\ \displaystyle \frac{1}{2} {b}_{12}&\displaystyle \frac{1}{2} {b}_{22}&\displaystyle &\displaystyle &\displaystyle \frac{1}{2} {b}_{2q}\\ \displaystyle &\displaystyle &\displaystyle &\displaystyle &\displaystyle \\ \displaystyle &\displaystyle &\displaystyle &\displaystyle &\displaystyle \\ \displaystyle \frac{1}{2} {b}_{1q}&\displaystyle \frac{1}{2} {b}_{2q}&\displaystyle &\displaystyle &\displaystyle {b}_{qq} \end{array} \right] \end{eqnarray*}

Stationary points can be obtained from Eq. (6);

(6) \begin{eqnarray*}{x}_{S}= \frac{1}{2} {\hat {\beta }}^{-1}b\end{eqnarray*}

If we substitute the stationary points in the main equation, Eq. (7) will be obtained;

\begin{eqnarray*}{\hat {Y}}_{S}={b}_{0}+{x}_{S}^{{^{\prime}}}b+{x}_{S}^{{^{\prime}}}\hat {B}{x}_{S} \end{eqnarray*}

(7) \begin{eqnarray*}{\hat {Y}}_{S}={b}_{0}+ \frac{1}{2} {x}_{S}^{{^{\prime}}}b\end{eqnarray*}

${\hat {Y}}_{S}$ is the value of the response variable estimated from the stationary point (Doğan & Okut, 2003; Schmidt & Launsby, 1992; Lucas, 1994; Lawson & Madrigal, 1994).

### Structure of stationary point (Canonical analysis)

When a quadratic equation is found to be adequate, the canonical analysis is applied to decide about the location and structure of stationary points in a quadratic equation. The signs of the eigenvalues obtained with the help of $\hat {B}$ matrix determine the stationary point structure. For this, it is possible to write a new equation containing canonical variables.

(8) \begin{eqnarray*}\hat {Y}={\hat {Y}}_{S}+\sum _{j=1}^{k}{\lambda }_{j}{W}_{j}^{2}\end{eqnarray*}

In Eq. (8), *λ*_1_, *λ*_2_, …, *λ*_*k*_ show the eigenvalues to be obtained from the vector $\hat {\beta }$, while *W*_1_, *W*_2_, …, *W*_*k*_ denote the “canonical variables”. It can be possible to understand the properties of the stationary points obtained with the help of Eq. (8).

(i) If *λ*_1_, *λ*_2_, …, *λ*_*k*_ eigenvalues are all negative, the stationary point represents the maximum point.

(ii) If *λ*_1_, *λ*_2_, …, *λ*_*k*_ eigenvalues are all positive, the stationary point shows the minimum point.

(iii) If the signs of *λ*_1_, *λ*_2_, …, *λ*_*k*_ eigenvalues are mixed, the stationary point indicates the “saddle point” (Doğan & Okut, 2003; Lawson & Madrigal, 1994; Alexander, 1997).

Initially, CCD and BBD experimental designs used in patient selection were created with the MINITAB package program (Tables 1 and 2). The statistical packages of Minitab® (Minitab Statistical Software, State College, Pennsylvania, USA) were used for routine data analysis and for both design and analysis of the central composite (CCD) and the Box–Behnken design (BBD).

**Table 1:** Central composite trial design.

StdOrder	RunOrder	PtType	Blocks	Age	HOMA	BMI
7	1	0	1	0	0	1
5	2	0	1	0.0	0.0	1
6	3	0	1	0.0	0.0	1
10	4	1	1	−1.0	1.0	2
14	5	0	1	0.0	0.0	2
3	6	1	1	−1.0	1.0	1
9	7	1	1	1.0	−1.0	2
1	8	1	1	−1.0	−1.0	1
2	9	1	1	1.0	−1.0	1
13	10	0	1	0.0	0.0	2
11	11	1	1	1.0	1.0	2
8	12	1	1	−1.0	−1.0	2
4	13	1	1	1.0	1.0	1
12	14	0	1	0.0	0.0	2
25	15	−1	2	0.0	1.4	2
23	16	−1	2	1.4	0.0	2
18	17	−1	2	0.0	1.4	1
22	18	−1	2	−1.4	0.0	2
27	19	0	2	0.0	0.0	2
26	20	0	2	0.0	0.0	2
17	21	−1	2	0.0	−1.4	1
19	22	0	2	0.0	0.0	1
16	23	−1	2	1.4	0.0	1
20	24	0	2	0.0	0.0	1
24	25	−1	2	0.0	−1.4	2
28	26	0	2	0.0	0.0	2
21	27	0	2	0.0	0.0	1
15	28	−1	2	−1.4	0.0	1

**Table 2:** Box-Behnken trial design with three factors.

StdOrder	RunOrder	PtType	Blocks	Age	HOMA	BMI	BBD
16	1	1	2	66.00	65.00	45.00	1
21	2	1	2	18.35	35.00	45.00	2
18	3	0	2	42.18	50.00	23.75	1
13	4	1	2	66.00	35.00	2.50	1
19	5	1	2	66.00	35.00	2.50	2
20	6	1	2	18.35	65.00	2.50	2
24	7	0	2	42.18	50.00	23.75	2
23	8	0	2	42.18	50.00	23.75	2
15	9	1	2	18.35	35.00	45.00	1
17	10	0	2	42.18	50.00	23.75	1
14	11	1	2	18.35	65.00	2.50	1
22	12	1	2	66.00	65.00	45.00	2
7	13	1	1	18.35	35.00	2.50	2
12	14	0	1	42.18	50.00	23.75	2
5	15	0	1	42.18	50.00	23.75	1
3	16	1	1	66.00	35.00	45.00	1
6	17	0	1	42.18	50.00	23.75	1
4	18	1	1	18.35	65.00	45.00	1
11	19	0	1	42.18	50.00	23.75	2
9	20	1	1	66.00	35.00	45.00	2
8	21	1	1	66.00	65.00	2.50	2
1	22	1	1	18.35	35.00	2.50	1
2	23	1	1	66.00	65.00	2.50	1
10	24	1	1	18.35	65.00	45.00	2
32	25	0	3	42.18	50.00	23.75	1
28	26	-1	3	42.18	74.50	23.75	1
37	27	-1	3	42.18	50.00	-10.95	2
35	28	-1	3	42.18	25.51	23.75	2
39	29	0	3	42.18	50.00	23.75	2
40	30	0	3	42.18	50.00	23.75	2
30	31	−1	3	42.18	50.00	58.45	1
36	32	−1	3	42.18	74.50	23.75	2
38	33	−1	3	42.18	50.00	58.45	2
33	34	−1	3	3.27	50.00	23.75	2
26	35	−1	3	81.08	50.00	23.75	1
34	36	−1	3	81.08	50.00	23.75	2
25	37	−1	3	3.27	50.00	23.75	1
31	38	0	3	42.18	50.00	23.75	1
29	39	−1	3	42.18	50.00	−10.95	1
27	40	−1	3	42.18	25.51	23.75	1

### Central composite experimental design

The Central Composite experimental design (CCD) is one of the most popular methods for generating a quadratic response level model. The CCD consists of a combination of 2^*k*^ two-level factorial experiments with *2k* number of axis points or star points, where “*k*” is the number of factors. It also contains *n*_*c*_** center points. The factors in the model should be at least two levels. The placement of the axis points in the experimental design [set-up] is given in Table 1. While the main effects of the quadratic model to be established and first-order interaction effects are obtained from 2^*k*^ experiments, the curvilinearity of the system is tested with the help of the center points and the quadratic terms in the model are estimated with the help of the axis points (Alexander, 1997; Doğan & Okut, 2003; Schmidt & Launsby, 1992; Lawson & Madrigal, 1994; Özler, 2006).

### Box-Behnken experimental design

These experimental design, introduced by Box and Behnken in 1980, are an effective method for constructing the quadratic response surfaces model. It is a method built on balanced incomplete block experiments. The factors to be included in the model must be at least three levels. If we try to explain the structure of the experiment with the help of a experimental design with three factors; in the Box-Behnken experimental design, while the value of one of the factors is fixed at the central value, the combinations for all levels of other factors are applied (Alexander, 1997; Doğan & Okut, 2003; Myers & Montgomery, 2002; Montgomery, 2001; Tekindal et al., 2012). As can be seen in Table 2, firstly the level of C factor is fixed, the combinations of all levels of A and B factors are applied and then the same operations are applied by fixing the levels of B and A factors in the center, respectively. In the last columns of the design matrix, there are the center point values.

### The smallest is the best

The aim is to reach the minimum response variable. When the aim is to minimize the response variable, the target value for the Taguchi response variable is treated as if it is zero. The squared loss function is defined as ${E}_{2}{ \left( y-0 \right) }^{2}$.

(9) \begin{eqnarray*}SN{R}_{s}=-10\,\log \nolimits \,\sum _{i=1}^{n} \frac{{y}^{2}}{n} \end{eqnarray*}

The above ratio is a ratio used to find *x* value that gives the levels of the control variables which minimize the expected squared loss error *E*_2_*y*^2^**.

${\mathop{\sum }\nolimits }_{i=1}^{n}$**:** the sum of the values in the outer zone of *n* response variables. The SNR value will be calculated for each inner zone point.

### The largest is the best

The aim is to reach the maximum response variable. It is handled in a similar way to “the smallest is the best” case. The value of $ \frac{1}{y} $ is substituted for the value of *y* in Equation 8.2 [Eq. (9)].

The expected squared loss error is obtained as follows;

(10) \begin{eqnarray*}{E}_{2}{ \left( \frac{1}{y} \right) }^{2}\end{eqnarray*}

and

(11) \begin{eqnarray*}SN{R}_{s}=-10\log \nolimits \sum _{i=1}^{n} \frac{1/{y}^{2}}{n} \end{eqnarray*}

### The target is the best

In this method, the aim to find *χ* value that gives the levels of the control variables that make the response variable the target value. In this case, two different SNRs are considered. Which SNR to use depends on the structure of the system. If the mean and variance of the response variable can be changed independently, one or more response variables can be used to remove the bias, according to Taguchi. These adjustment [manipulated] variables allow the researcher to change the mean without changing the variance. This analysis has two stages. First, the adjustment factors that enable the response variable to reach the target value are selected, and then the levels of other control factors that minimize the SNR value are determined. In this case, the expected squared loss error is obtained as follows (Tekindal et al., 2012; Tekindal & Kaymaz, 2018; Tekindal et al., 2014; Bayrak, Özkaya & Tekindal, 2010);

(12) \begin{eqnarray*}{E}_{2}{ \left( y-t \right) }^{2}\end{eqnarray*}

and

(13) \begin{eqnarray*}SN{R}_{T1}=-10\log \nolimits {s}^{2}\end{eqnarray*}

*s*^2^** is the sample variance calculated over the experimental points located in the outer zone of the cross zone, and determined with the following equation.

(14) \begin{eqnarray*}{s}^{2}= \frac{\sum _{i=1}^{n}{ \left( {y}_{i}-\bar {y} \right) }^{2}}{n-1} \end{eqnarray*}

When the factor levels that maximize the SNR value are applied, the value of the response variable will reach the target value.

For cases where the standard error of the response variable is related to the mean values, a different SNR value is proposed. This is a value that can be best applied in cases where the relationship is linear (Tekindal et al., 2012; Tekindal & Kaymaz, 2018; Tekindal et al., 2014; Bayrak, Özkaya & Tekindal, 2010).

(15) \begin{eqnarray*}SN{R}_{T2}=-\log \nolimits \left( \frac{{\bar {y}}^{2}}{{s}^{2}} \right) \end{eqnarray*}

### Data set

From the results of these experiments, firstly, the point belonging to the factor levels providing the highest efficiency is estimated; then it is tried to determine the point in question (the combination that provides the highest efficiency), with the way to reach where the actual optimum point is or by making the steepest increase from the second-order [quadratic] by using the coefficients of the first-order response surface function of the first experimental results (the steepest ascent method).

The study data belong to 40 obese patients who lost weight through the Bariatric Surgery between February 2015 and December 2016. The values of BMI, Age and HOMA for the obese patients who lost weight through the Bariatric Surgery were evaluated in three categories and at three levels. Data from a subset of patients from Altin et al., published in 2016, were used in the current study (Altin et al., 2018). “Verbal informed consent” was obtained from the participants. The response variable was determined as the ΔEFT. The experimental design was set up before starting the study, and the study data were identified in accordance with the experimental design. Informed consent was obtained from the study participants.

In the study, after the CCD and BBD designs were determined, patients were selected for 40 subjects who were admitted to the hospital and met the relevant criteria. The age of 40 subjects is 40.45 ± 11.30 minimum age 18 and maximum 66 years. Also, the BMI is 45.95 ± 7.51 minimum 35.06, maximum 64.47. The pre-experimental HOMA values are 8.09 ± 6.52 minimum 2.72 and maximum 38.40. In the study, weights were measured in kg, 2 digits after the comma, before and after the patients were only wearing surgical gowns. Within the scope of the study per protocol, all patients were followed during the 180-day follow-up period and the final measurements were taken. The study was carried out by providing all the assumptions of CCD and BBD experimental designs. The target is best algorithm was used in our study.

The division into three categories for age, BMI and HOMA values in Table 3 was made by field experts and co-authors within the framework of clinical observation.

**Table 3:** Data structure for trial design with three factors.

**Age**	**BMI**	**HOMA**	Δ**EFT**
18–35	35-45	2.5–15	2.473
		15.1–35	1.75
		35.1–45	2.5
	45.1–55	2.5–15	4.5
		15.1–35	2.5
		35.1–45	2.14
	55.1–65	2.5–15	2
		15.1–35	2.5
		35.1–45	2.71
36–45	35–45	2.5–15	1.72
		15.1–35	2.5
		35.1–45	2
	45.1–55	2.5–15	2
		15.1–35	3.5
		35.1–45	2.3
	55.1–65	2.5–15	4
		15.1–35	2.14
		35.1–45	3.4
45–66	35–45	2.5–15	1.83
		15.1–35	2.21
		35.1–45	2.37
	45.1–55	2.5–15	1.98
		15.1–35	1
		35.1–45	2
	55.1–65	2.5–15	2
		15.1–35	2.02
		35.1–45	2.42

## Findings

Forty obese patients who lost weight by the bariatric surgery between February 2015 and December 2016 were included in the study. The values of BMI, Age, and HOMA were evaluated in three categories and at three levels, and the ΔEFT was chosen as the response variable. The experimental design [set-up] was planned before the study. The study data were collected in accordance with the experimental design. The informed consent was obtained from the study participants.

First of all, 3^3 ^ experimental designs [set-ups] were determined by CCD [design] and the contours and response plots were drawn according to the steepest ascent/descent condition.

While planning the CCD design; the three factors were set up with α = 1.633, six axial points, four central points, and two axial center points.

### Measurement of epicardial fat thickness

EFT can be identified as the echo-free space between the free [outer] wall of the myocardium and the visceral layer of the pericardium. It is measured by the standard transthoracic 2D echocardiography (Vivid S5 ultrasound machine, GE, Healthcare, Horten, Norway) in the parasternal long-axis views of three cardiac cycles at the end of the diastole and perpendicular to the right ventricular free wall as previously described (Altun, 1998; Kul, 2004; Mead & Pike, 1975). The same investigators, who were blinded to all clinical data of the patients, performed all measurements. After that, the data were digitally stored and reviewed by a senior echocardiographer in order to avoid the inter-reader variability.

As can be seen in Table 4, the interaction effects of variables of Age and BMI, Age and HOMA, and BMI and HOMA [on ΔEFT] were found to be significant (*p*; 0.041, 0.031 and 0.026, respectively). According to these results, the optimum ΔEFT combinations were determined by drawing the contour and response plots, and the model was expressed as specified in Eq. (16). The *R*^2^** value of the model was found to be 87.75%.

(16) \begin{eqnarray*}\Delta \mathrm{EFT}=2.03+0.0198~\mathrm{Age}+0.013~\mathrm{BMI}+0.0078~\mathrm{HOMA}-0.000481\nonumber\\\displaystyle ~\mathrm{Age}\ast ~\mathrm{Age}-0.00028~\mathrm{BMI}\ast ~\mathrm{BMI}-0.000282~\mathrm{HOMA}\ast ~\mathrm{HOMA}+0.000168\nonumber\\\displaystyle ~\mathrm{Age}\ast ~\mathrm{BMI}+0.000055~\mathrm{Age}\ast ~\mathrm{HOMA}+0.000221~\mathrm{BMI}\ast ~\mathrm{HOMA}\nonumber\\\displaystyle ~\mathrm{The}~{R}^{2}~\mathrm{of~ this~ model~ was}~0.88\end{eqnarray*}

**Table 4:** Results of CCD trial design analysis.

	Sd	Adjusted Std. error	Adjusted average of squares	F	*p*
Model	11	5.495	0.500	4.65	0.002
Block	2	2.921	1.461	4.90	0.012
Linear	3	1.314	0.438	4.57	0.041
Age	1	0.925	0.925	4.20	0.005
BMI	1	0.030	0.030	6.04	0.049
HOMA	1	0.359	0.359	4.47	0.014
Quadratic form	3	1.185	0.395	4.51	0.085
Age*Age	1	1.014	1.014	4.32	0.084
BMI*BMI	1	0.053	0.053	4.07	0.001
HOMA*HOMA	1	0.213	0.213	4.28	0.013
Interaction effect	3	0.075	0.025	5.03	0.042
Age*BMI	1	0.029	0.029	4.04	0.041
Age*HOMA	1	0.006	0.006	2.01	0.031
BMI*HOMA	1	0.040	0.040	4.05	0.026
Error	8	6.161	0.770		
Adjustment	5	6.161	1.232	*	*
Net error	3	0.000	0.000		
Total	19	11.656			

**Figure 1:**
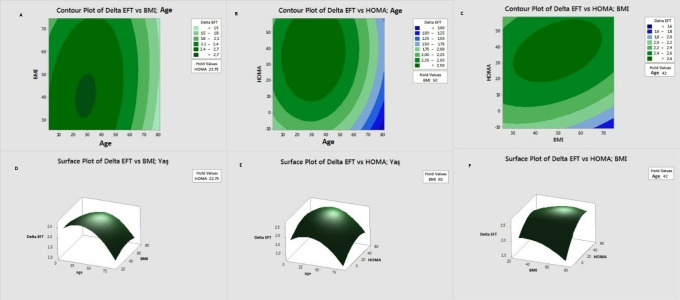
Contour and response surface graphs for CCD design.

When Fig. 1 was examined, as a result of CCD analysis, the optimum ΔEFT was determined as ΔEFT = 2.571, where Age = 30.52, BMI = 45.30, HOMA = 34.62.

While planning the BBD design, it was established as three factors and three central points.

When Table 5 was examined, the interaction effects of variables of Age and BMI, Age and HOMA, and BMI and HOMA [on ΔEFT] were found to be significant (*p*; 0.043, 0.040 and 0.022, respectively). According to these results, the optimum ΔEFT combinations were determined by drawing the contour and response plots, and the model was expressed as specified in Eq. (17). The *R*^2^** value of the model was found to be 91.27%.

(17) \begin{eqnarray*}\Delta \mathrm{EFT}=-7.72+0.1718~\mathrm{Age}+0.245~\mathrm{BMI}+0.0599~\mathrm{HOMA}-0.001746\nonumber\\\displaystyle ~\mathrm{Age}\ast \mathrm{Age}-0.00166~\mathrm{BMI}~\ast \mathrm{BMI}-0.000766~\mathrm{HOMA}\ast \mathrm{HOMA}-0.000653\nonumber\\\displaystyle ~\mathrm{Age}\ast \mathrm{BMI}+0.000173~\mathrm{Age}\ast \mathrm{HOMA}-0.000690~\mathrm{BMI}\ast \mathrm{HOMA}\nonumber\\\displaystyle {R}^{2}~\mathrm{of~ the~ model~ was~ 0.91}\end{eqnarray*}

**Table 5:** Results of BBD trial design analysis.

	Sd	Adjusted Std. error	Adjusted average of squares	F	*p*
Model	9	7.087	0.788	4.12	0.012
Linear	3	2.351	0.784	4.10	0.027
Age	1	0.053	0.053	4.21	0.045
BMI	1	2.247	2.247	8.89	0.031
HOMA	1	0.050	0.050	4.20	0.075
Quadratic form	3	4.291	1.430	4.66	0.046
Age*Age	1	3.736	3.736	14.78	0.012
BMI*BMI	1	0.517	0.517	4.04	0.012
HOMA*HOMA	1	0.442	0.442	4.75	0.043
Interaction effect	3	0.446	0.149	4.59	0.049
Age*BMI	1	0.221	0.221	4.87	0.043
Age*HOMA	1	0.031	0.031	4.12	0.040
BMI*HOMA	1	0.194	0.194	4.77	0.022
Error	5	1.264	0.253		
Adjustment	3	1.264	0.421		
Net error	2	0.000	0.000		
Total	14	8.351			

**Figure 2:**
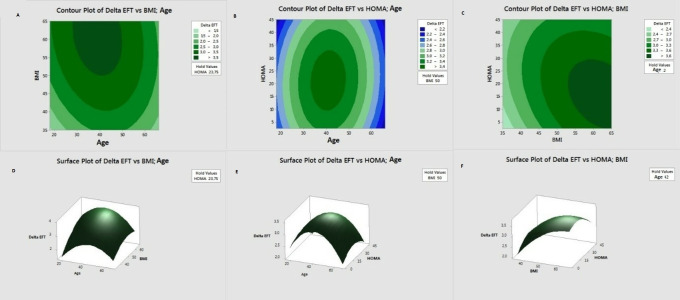
Contour and response surface graphs for BBD experiment design.

When Fig. 2 was examined; as a result of the BBD analysis, the optimum ΔEFT = 3.756 was determined, where Age =38.36, BMI =63.18, HOMA =14.95.

**Figure 3:**
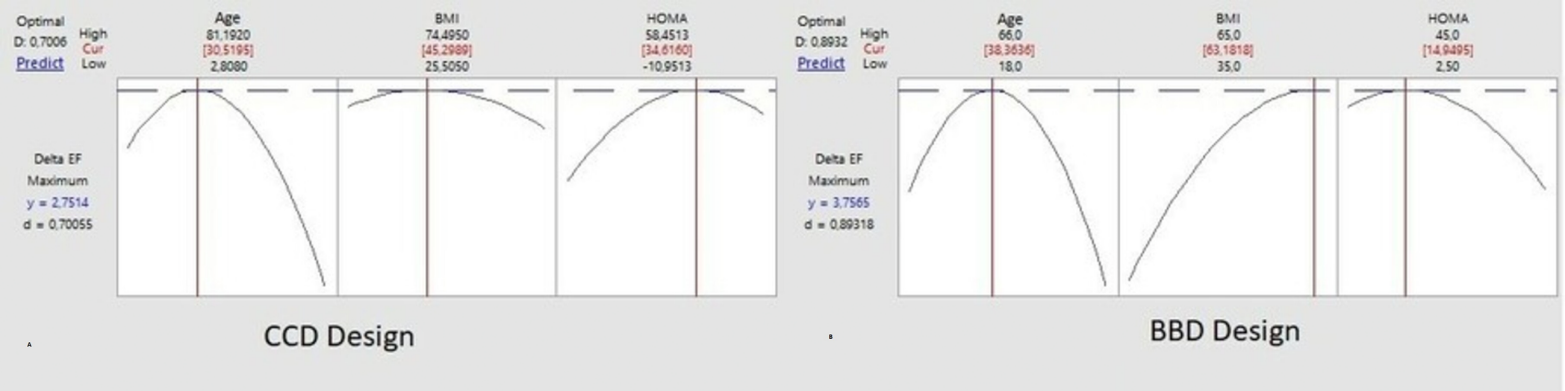
CCD and BBD design optimal point.

Figure 3 shows clear optimal differences between CCD and BBD designs.

## Discussion and Conclusion

Jeong et al. (2007) included 202 patients and found a significant correlation between EFT thickness measured by the echocardiography and the severity of coronary artery disease, suggesting that it could be used in the risk stratification in patients with coronary artery disease. Ahn et al. (2008) measured EFT thickness with the echocardiography for 527 patients who underwent the coronary angiography for the first time and they reported a relationship between EFT thickness and the activation and prevalence of coronary artery disease. In addition, the relationship between the epicardial fat thickness and the insulin resistance and inflammation was also shown in the same study. Ulucan et al. (2015) showed in their study that the increase in the volume of EFT was significantly associated with the increase in the frequency of long-term major adverse cardiovascular events. In our study, the relationship between EFT thickness and age and obesity was found. There are studies showing the relationship between atherosclerosis risk and advanced age and obesity (Aballay et al., 2013; Chang et al., 2013). Considering the results of this study, it is an expected result that EFT thickness in elderly and obese individuals is higher. In our study, there was no significant difference between the groups in terms of the cholesterol value. However, the statin use was more in the group with higher EFT thickness. This finding suggests that the patients in the group with high EFT thickness were considered to have a higher cardiovascular risk, and therefore the statin might have been started.

Today, the obesity surgery is frequently applied in developed countries around the world. Among the surgical procedures, the most preferred method is the sleeve gastrectomy. With weight loss after the bariatric surgery, a significant improvement has been observed in the chronic diseases such as hypertension, hyperlipidemia, atherosclerosis, and diabetes mellitus. This improvement is mainly related to breaking the insulin resistance. However, not all patients benefit equally from these surgical operations. Some patients benefit greatly from them, while others get more limited benefit. If it is known which patient group benefits most, these patients can be directed to the surgery in an earlier time. However, this requires advanced statistical analysis. Epicardial adipose tissue is a visceral adipose tissue, and its measurement is a good marker for these chronic diseases. The reduction in epicardial adipose tissue is a reliable parameter that can be used to determine which patient group benefits most (Rosenthal et al., 2012; Iacobellis et al., 2003; Eroglu et al., 2009).

One of the aims of the response surface studies is to identify an appropriate function (or model) that will determine the relationship between the response variable and the input variables in order to accurately predict the future values of the response variable. Another one is to investigate the maximum or the minimum response value depending on the type of the problem and to determine the values of the input variables that can provide this value. Finally, they contribute to understanding the mechanism underlying a response system. As a result of CCD analysis, the optimum ΔEFT = 2.571 was determined when Age = 30.52, BMI = 45.30, HOMA = 34.62. As a result of the BBD analysis, the optimum ΔEFT = 3.756 was determined when Age = 38.36, BMI = 63.18, HOMA = 14.95. The optimum ΔEFT was modeled with the Contour and Response Surface plots. According to the results of the analysis, it was revealed that BBD analysis generated more positive results for the optimum ΔEFT than CCD, and also the optimum combinations of Age, BMI and HOMA were determined in order to reach the maximum ΔEFT.

The body mass index and waist circumference are the widely accepted measurements of generalized adiposity; however, they are poor indicators for the visceral obesity. It is well-known that the visceral adipose tissue accumulation is associated with the subclinical atherosclerosis and increases the cardiovascular risk more strongly than the generalized adiposity (Chang et al., 2013). Emerging data have suggested that ΔEFT is a reliable method to estimate the visceral adiposity and strongly correlates with the cardiometabolic risk factors independent of overall adiposity (Natale et al., 2009; Nelson et al., 2011). ΔEFT ≥5 mm was found to be associated with a higher incidence of detectable carotid atherosclerosis (Rabkin & Campbell, 2015). In addition, ΔEFT may be a modifiable factor for CVD or a target to modify the cardiovascular risks (Fried et al., 2014). Early atherosclerotic structural changes, including ΔEFT and the carotid intima-media thickness may be reversed or improved by sustained weight loss following LSG for the asymptomatic obese patients (Güvenç et al., 2006).

Patients are generally operated according to the standard bariatric surgical indications in the guidelines (Jeong et al., 2007; Ahn et al., 2008). However, which patients get more cardiovascular benefits following the bariatric surgery is uncertain. Our results may prove to be helpful in addressing this question. A short follow-up period is an important limitation of our study. Randomized, prospective, and large-scale further studies are required to confirm our results.

The response variable was the decrease of epicardial fat thickness as measured by echocardiography. This parameter is a one-dimensional estimate (thickness) of a three-dimensional mass of fat (cm^3^). Age and bodyweight are pure variables, but BMI = body weight/ (body lengths) ^2^ [kg/m^2^] and HOMA-IR are indexes and in fact are hybrid variables based on the body weight and lengths and fasting blood insulin and glucose concentration, respectively.

In our patient population the BBD analysis generated better results for the optimum ΔEFT than CCD for specific values for Age, BMI and HOMA.

In conclusion, our study suggests that the weight loss after the bariatric surgery improves the cardiac functions for morbidly obese patients. Our findings showed that there was no negative change in the systolic and diastolic functions of the patients, and the myocardial performance index, which evaluates the systolic and diastolic functions together, improved. At the same time, the positive changes such as decrease in the intracardiac chronic pressure and volume load, significant decrease in the systolic and diastolic blood pressure as well as heart rate and decrease in fasting blood glucose contribute to the current recovery.

## Supplemental Information

10.7717/peerj.11831/supp-1 Supplemental Information 1 Raw Data

## Additional Information and Declarations

### Data Availability

The following information was supplied regarding data availability: Raw data are available in the Supplemental Files.

### Clinical Trial Registration

The following information was supplied regarding Clinical Trial registration: This study was approved by Baskent University Institutional Review Board and Ethics Committee (Approval number: KA16/281).
